# Cholangioscope-assisted endoscopic retrograde appendicitis therapy for occult chronic appendicitis

**DOI:** 10.1055/a-2335-6826

**Published:** 2024-07-29

**Authors:** Qianlong Li, Ting Qin, Tianyu Liu, Linlin Chen, Biao Jiang

**Affiliations:** 1 529706 Fourth Department, Digestive Disease Center, Suining Central Hospital, Suining, China; 2 529706 Infectious Diseases Department, Suining Central Hospital, Suining, China

In recent years, endoscopic retrograde appendicitis therapy (ERAT) has been widely used in the treatment of acute uncomplicated appendicitis, especially with the assistance of cholangioscopy 1 2 ; however, the diagnosis and treatment of chronic appendicitis has always been a challenge. Chronic appendicitis is often overlooked owing to atypical symptoms and unclear imaging changes 3 . We report a case of recurrent unexplained lower right abdominal pain. Through cholangioscope-assisted ERAT, the patient was diagnosed as having chronic appendicitis with a pinhole-like stenosis and suppuration, adequate drainage was performed, and the abdominal pain was completely relieved.

A 35-year-old woman presented with recurrent lower right abdominal pain for 6 months. Computed tomography (CT) showed her appendix to be normal. After the patient had given informed consent, a cholangioscope-assisted ERAT procedure was performed ( Video 1 ). Colonoscopy showed no abnormalities in the appendix. A cholangioscope was inserted into the appendiceal cavity, where scattered mucosal congestion and a pinhole-like stenosis were observed ( Fig. 1 **a** ). A guidewire was inserted through the stenosis, releasing a large amount of pus that was seen to flow out through the stenosis ( Fig. 1 **b** ). The anterior end of the cholangioscope was used to dilate the stenosis ( Fig. 1 **c** ). The cholangioscope was successfully passed through the stenosis to the blind end of the appendix and, after washing out the pus, a biliary plastic stent was placed ( Fig. 2 ). Postoperatively, the patientʼs refractory abdominal pain completely disappeared.

**Fig. 1:**
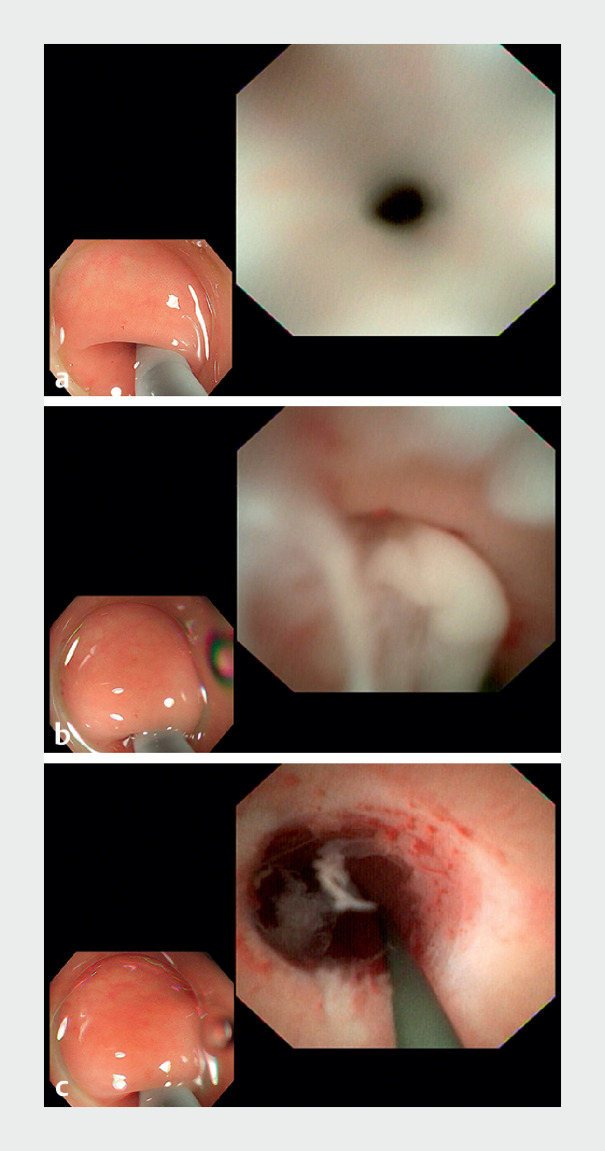
Cholangioscopic view of the appendix showing: **a** a pinhole-like stenosis near the blind end of the appendix; **b** a large amount of pus flowing from the stenosis after insertion of the guidewire; **c** the anterior end of the cholangioscope successfully dilating the stenosis with guidewire guidance.

**Fig. 2:**
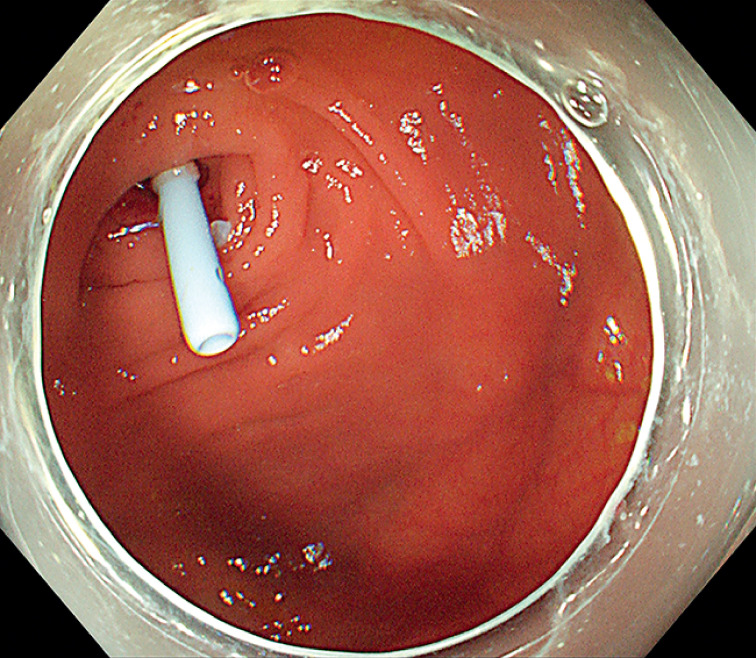
Colonoscopic view showing a biliary plastic stent that was successfully placed into the appendix after the pus had been washed out.

Cholangioscope-assisted endoscopic retrograde appendicitis therapy is performed for occult chronic appendicitis with a pinhole-like stenosis and suppuration.Video 1

At follow-up after 7 months, the patient reported having had no further abdominal pain. The stent was removed colonoscopically. Repeat examination of the appendix with a cholangioscope showed the mucosa was smooth and the stenosis had disappeared ( Fig. 3 ).

**Fig. 3:**
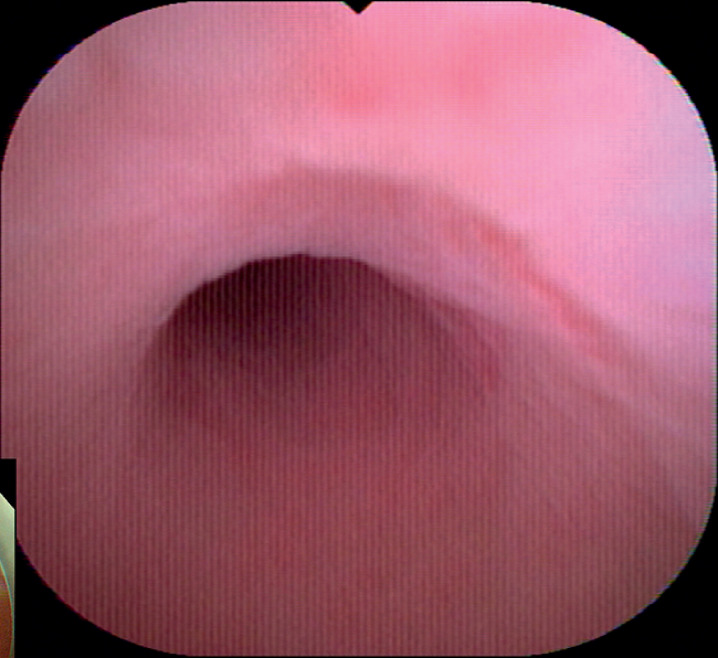
Follow-up cholangioscopic appearance after 7 months showing smooth appendiceal mucosa, with the stenosis no longer visible.

Cholangioscope-assisted ERAT may be a very effective and safe way to diagnose and treat occult chronic appendicitis.

Endoscopy_UCTN_Code_TTT_1AQ_2AF

## Notes

Endoscopy E-Videos https://eref.thieme.de/e-videos
*E-Videos* is an open access online section of the journal *Endoscopy* , reporting on interesting cases and new techniques in gastroenterological endoscopy. All papers include a high-quality video and are published with a Creative Commons CC-BY license. Endoscopy E-Videos qualify for HINARI discounts and waivers and eligibility is automatically checked during the submission process. We grant 100% waivers to articles whose corresponding authors are based in Group A countries and 50% waivers to those who are based in Group B countries as classified by Research4Life (see: https://www.research4life.org/access/eligibility/ ). This section has its own submission website at https://mc.manuscriptcentral.com/e-videos .
